# Recognition of Elder Abuse by Chinese Nursing Undergraduates: Evaluation by the Caregiving Scenario Questionnaire

**DOI:** 10.1093/geroni/igaa057.052

**Published:** 2020-12-16

**Authors:** Zhuzhu Wang, Jiayuan Zhuang, Qiaoxian Zhang, Xiaolan Lai, Qixi Liu, Baoyuan Xie, Qinmei Tang

**Affiliations:** 1 Fujian Medical University, Fuzhou, Fujian, China; 2 The First Affiliated Hospital of Fujian Medical University, Fuzhou, Fujian, China; 3 Ningde Mindong Hospital, Fu’an City, Fujian, China; 4 The Second Affiliated Hospital of Fujian Medical University, Fuzhou, Fujian, China; 5 Xiamen University affiliated second Hospital of Fuzhou, Fuzhou, Fujian, China

## Abstract

This study aimed to evaluate the recognition of elder abuse (EA) among nursing undergraduates in China and determine whether recognition is related to sociodemographic factors and education. We conducted a cross-sectional study with stratified random sampling, using the Caregiving Scenario Questionnaire of Chinese version (CSQ-CV). Questionnaires were disseminated to 343 nursing undergraduates ranged from 1st to 4th year at Fujian Medical University, China. The content validity of CSQ-CV is 0.97. 340 students (99.1%) effectively responded. 223(65.6%) of them identified trapping someone in an armchair; 108(31.8%) locking someone alone; and 3(0.9%) accepting someone was not clean as abusive. The majority correctly identified four out of five non-abusive (NA) items, while 210(61.8%) incorrectly identified camouflaging the door to prevent wondering outdoors. With respect to potential-abusive (PA) items, less than half of the students made right judgements. Only 30(8.8%) correctly identified not taking her to family gatherings; 46(13.5%) telling her only having breakfast after bathing; and 50(14.7%) hiding tablets in someone’s cereal or tea. Mann-Whitney and Kruskal-Wallis tests indicated no significance related to correct recognition of the three abusive items with sociodemographic factors and education. The students who were female, caring older adults at home or had detected EA cases were more likely to correctly recognize NA items (Z=-2.428,P=0.015;Z=-2.028,P=0.043;Z=-2.534,P=0.011). Besides, female students got higher scores of CSQ-CV (Z=-2.000, P=0.045). Nursing undergraduates’ recognition of EA, especially in neglect and PA are still at a low level in China. Education about EA in nursing undergraduates’ curriculum and training program should be encouraged.

